# Outpatient Management of Fever and Neutropenia in Low-risk Children with Solid Tumors: A Quality Improvement Initiative

**DOI:** 10.1097/pq9.0000000000000771

**Published:** 2024-09-25

**Authors:** Wallace Bourgeois, Jonathan Paolino, Riley Garland, Kevin Campbell, Francesca Alvarez-Calderon, A. Lindsay Frazier, Allison F. O’Neill, Maya Ilowite, Chris I. Wong

**Affiliations:** From the * Dana-Farber Cancer Institute/Boston Children’s Cancer and Blood Disorders Center and Harvard Medical School, Department of Pediatric Oncology, Boston, Mass.; † Division of Pediatric Hematology-Oncology, University Hospitals Rainbow Babies and Children’s Hospital, Cleveland, Ohio; ‡ Division of Hematology-Oncology, University Hospitals Seidman Cancer Center, Case Western Reserve University School of Medicine, Cleveland, Ohio.

## Abstract

**Background:**

Management of febrile neutropenia in pediatric oncology usually requires inpatient parenteral antibiotics after initial evaluation, but some patients at lower risk of sepsis could be safely managed outpatient. We describe a quality improvement project to increase outpatient management of fever and neutropenia.

**Methods:**

We designed a standardized algorithm for children with a solid tumor diagnosis and low risk for bacteremia. The aim was to achieve outpatient management for at least 80% of eligible patients within 20 months of project initiation. We used plan-do-study-act cycles to improve algorithm compliance, including optimizing medical record decision support, developing targeted educational materials and outreach, and restructuring outpatient processes to allow for close follow-up. We surveyed patients (age ≥12 y) and parents/caregivers to assess the impact of outpatient management.

**Results:**

The initiative led to 71% (n = 34) of eligible patients being managed as outpatients. Six percent (n = 2) of patients developed bacteremia, resulting in hospital admission. Fifteen of 26 parents/caregivers and five of 11 patients approached completed the survey. For the preferred setting of febrile neutropenia management, 83% of patients preferred to be home versus 40% of parents/caregivers. No patient expressed any of the three highest ratings in the question exploring fear regarding outpatient febrile neutropenia management versus 67% of parents/caregivers.

**Conclusions:**

Some children with a solid tumor diagnosis at low risk for bacteremia are safely managed for febrile neutropenia as outpatients. Targeted efforts to engage parents/caregivers early in this practice change are necessary for success.

## INTRODUCTION

Infection is the largest contributor to treatment-related mortality in pediatric cancer, accounting for up to half of all treatment-related mortality events.^1^ As such, children with fever and neutropenia receive parenteral antibiotics while admitted to the hospital. Several quality improvement (QI) initiatives have established processes to reduce time to antibiotics and risk of infections^2–4^ but less have focused on optimizing opportunities to safely treat as outpatients those at low risk for bacteremia during fever and neutropenia.^5^

Although some guidelines exist^6–9^ for outpatient management of fever and neutropenia, this is not yet the standard of care. The most appropriate cohort of patients at the lowest risk for fever and neutropenia complications is unknown. Importantly, the impact of this practice change on parents/caregivers/patients is not well understood.^10^ We developed a QI initiative to manage at least 80% of all eligible, low-risk patients with solid tumors as outpatients during episodes of fever and neutropenia within 20 months of project initiation. We sought a caregiver/patient perspective with this change.

## METHODS

### Context

We conducted this initiative within a large academic pediatric oncology practice from March 2021 to December 2022, the Dana-Farber/Boston Children’s Hospital Cancer and Blood Disorders Center. Historically, all patients with fever and neutropenia received inpatient management in the hospital for parenteral antibiotics until neutrophil recovery. This initiative proposed outpatient management of patients at low risk of bacteremia. It began within the solid tumor group [non-central nervous system (non-CNS)] as these patients usually experience faster neutrophil recovery than children with hematologic malignancies, and they are at lower risk of bacteremia.^11^ Programmatic leadership supported the implementation of this change.

**Fig. 1.**
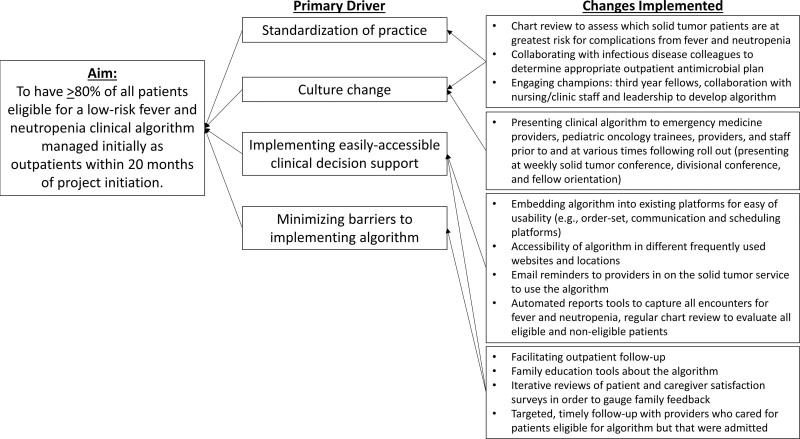
Key driver diagram with specific aim, primary drivers, and changes implemented to drive practice change for management of fever and neutropenia in low-risk patients with solid tumors.

We performed a literature review on outpatient fever and neutropenia management^12^ and retrospectively reviewed the charts of patients with non-CNS solid tumors managed with fever and neutropenia (and no identified source of infection) at our hospital from January 1 to July 1, 2019 (**Fig. 1, Supplemental Digital Content 1,** http://links.lww.com/PQ9/A602). In this group of 35 patients; there were three episodes of bacteremia. The average time to neutrophil count recovery (≥500 cells/mm^3^) was 3.8 days. We also reviewed all patients with a non-CNS solid tumor diagnosis who had experienced a central line infection from 2018 to 2019 (n = 12). Amongst the episodes of bacteremia, eight occurred in patients with high-risk neuroblastoma, and two occurred in patients less than 1 year of age.

### Intervention

**Fig. 2.**
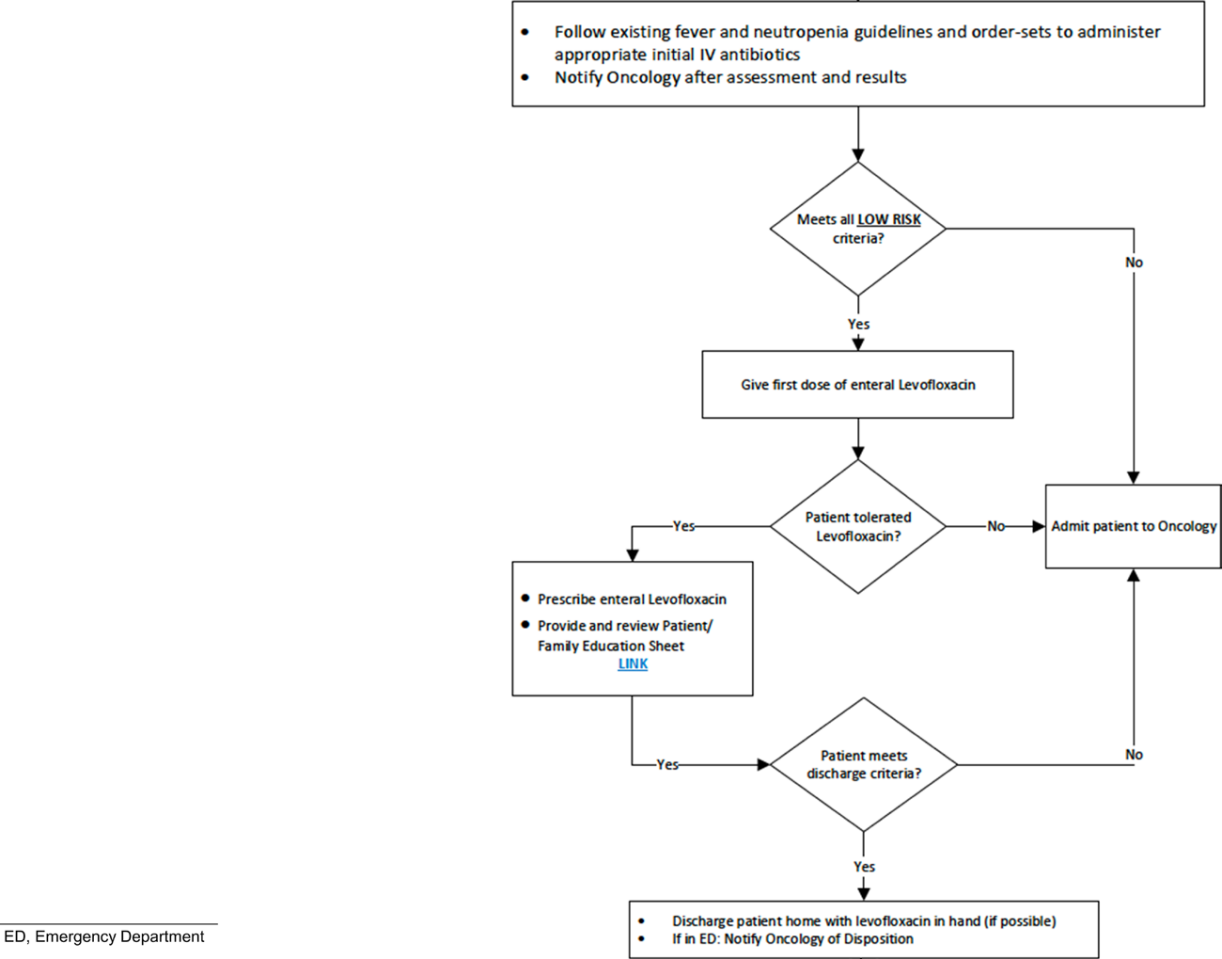
Short version of fever and neutropenia algorithm for management in the outpatient setting of patients with solid tumors at low risk of bacteremia.

We convened a multidisciplinary team consisting of pediatric solid tumor faculty, nurses, pharmacists, and fellows to define the SMART (specific, measurable, attainable, realistic, time-bound) aim, carry out the QI intervention, and identify drivers of success (Fig. 1). The graduating class of pediatric hematology-oncology fellows led this as part of their QI experience during training. With the data from the retrospective reviews, and in partnership with colleagues from the emergency room (ER), pharmacy, and infectious disease, a clinical algorithm was developed (Fig. 2, **Fig. 2, Supplemental Digital Content 2,** http://links.lww.com/PQ9/A603). We iterated the algorithm using feedback from other key stakeholders, including physician leaders/faculty, clinic nurses, fellows, and patient’s parents/caregivers until reaching consensus. Specifically, members of the pediatric patient and family advisory council provided feedback. We discussed the algorithm with parents/caregivers to ascertain their perception of the proposed changes. Departmental websites had new updates to include the final clinical algorithm. We also linked the algorithm to our fever and neutropenia order-set in the electronic medical record (EMR) and the platform for scheduling outpatient visits and distributing them to clinical teams electronically and on paper.

We created a new algorithm for outpatient management as a guideline after the initial management of fever and neutropenia. The previously established fever and neutropenia guidelines require obtaining blood cultures and a complete blood count with differential and promptly administering parenteral antibiotics to all patients based on initial risk of bacteremia (high versus standard) (**Table 1, Supplemental Digital Content 3,** http://links.lww.com/PQ9/A604).

After the initial management and administration of parenteral antibiotics, a subset of children with non-CNS solid tumors could then be considered low risk for fever and neutropenia management and eligible for outpatient management using the newly developed algorithm if they met all criteria: non-CNS solid tumor diagnosis, >1 year of age, central access with a port-a-cath, ability to follow-up within 24–48 hours to clinic, and to tolerate oral levofloxacin. Exclusion criteria were (1) active clinical issue (eg, signs or symptoms of sepsis or severe mucositis), (2) clinical risk factor (eg, surgery within the last 2 wk, levofloxacin allergy, high-risk criteria), or (3) social risk factor (eg, unreliable social situation or first cycle of chemotherapy). If the patient met inclusion but not exclusion criteria for outpatient management, they received a first dose of oral levofloxacin in the ER to assess tolerability, followed by a prescription sent to their local pharmacy. The evaluating ER provider and the pediatric oncology provider on-call would discuss the patient’s case and decide on discharge and outpatient management. Then, the ER provided education sheets to the patient/caregiver, and the oncology provider arranged follow-up. Patient follow-up occurred in the oncology clinic 24–48 hours after ER discharge unless the patient was in the ER on a Friday. In this case, the oncology on-call team would contact the patient over the weekend to assess clinical status and arrange a follow-up for Monday. Patients went to a local laboratory center or our clinic for a blood draw at least twice weekly. When the patient was neutropenic in the outpatient setting, a clinic triage nurse contacted the patient/caregiver every day until neutrophil count recovery ≥500 cells/mm^3^ to assess for any complications or criteria for admission. Admission criteria were positive blood culture, inability to tolerate oral antibiotics, fever lasting 5 or more days, or signs of severe illness or localized infection. Upon neutrophil count recovery, patients discontinued oral levofloxacin. In coordination with infectious disease colleagues, we decided to use levofloxacin until neutrophil recovery due to its expanded coverage, bioavailability, ease of administration, and overall tolerability.

To implement outpatient fever and neutropenia management, we used plan-do-study-act cycles targeted to increase provider and patient adherence to the clinical algorithm. We collected and analyzed data monthly, triggering tests of change. Changes included embedding the algorithm into the ER fever and neutropenia management order-set in the EMR; optimizing clinical decision support in the order-set to delineate parenteral antibiotic followed by a one-time dose of oral levofloxacin and directions to prescribe subsequent doses; adapting scheduling forms in our existing request platform to allow for follow-up visits per the algorithm; developing a job aid to standardize follow-up of patients by triage nurses; and targeted educational outreach including the development of educational materials to all involved stakeholders.

Before “Go-Live,” the QI team presented the algorithm to all pertinent staff, including pediatric hematology-oncology and ER staff/trainees, inpatient and outpatient nurses, clinic staff, and schedulers. To educate patients and parents/caregivers on this practice change, we engaged nurses as partners to review the expected change during their hospital stays and routine outpatient visits. We developed a one-page education sheet to help patients and parents/caregivers prepare for this practice change. Staff education occurred at least 30 days before Go-Live.

In the first months after launching this initiative, team members contacted on-call providers ahead of their shifts to remind them of the algorithm. A month after Go-Live, the QI team re-educated the providers on the algorithm. We used informal feedback from providers who did not use the algorithm to understand barriers. Subsequent educational sessions were done to increase engagement based on the results observed regarding adherence to the algorithm.

### Impact of Outpatient Management on Patients and Families

We developed surveys to assess the impact and perception of outpatient management on patients and parents/caregivers. We used relevant fever and neutropenia literature wherein caregiver and/or patient feedback was elicited to guide the development of caregiver and patient preference surveys.^5^ The survey had 21 questions for parents/caregivers, with a similar 19-question survey for patients. Examples of questions included asking about the quality of sleep at home, ability to keep up with schoolwork, ability to keep up with housework (parents/caregivers only), appetite, administering/taking antibiotics by mouth, time with siblings, confidence in care, fear, preference of being managed in the inpatient versus outpatient setting (at home), and open narrative comments. A 5-point Likert scale (1 = not at all, 5 = very) assessed fear and confidence in doing this outpatient management at home. We asked patients and parents/caregivers to evaluate these parameters as if admitted to the hospital instead of their experienced outpatient management. We translated surveys into Arabic, Mandarin, and Spanish. For willing caregivers, we sent a survey link via email through RedCap. If the patient was at least 12 years, we sought permission to send a similar survey to the patient.

### Study of the Intervention

We collected data prospectively from March 2021 to December 2022. A report identified all patients evaluated for fever and neutropenia in the clinic or ER and distributed to team members every week. Abstraction from the EMR included clinical characteristics, initial management, disposition, and clinical follow-up during the intervention. In addition, we also reviewed the records of patients admitted to the hospital but potentially eligible for the algorithm. We contacted their primary team to determine whether the patient met the eligibility criteria and, if so, why they did not pursue outpatient management.

### Measures

The primary outcome measure was the percentage of eligible patients with fever and neutropenia managed as outpatients. As a balance measure, we determined the percentage of patients that failed outpatient management, defined as patients who were initially outpatient and then were admitted to the hospital due to a complication of fever and neutropenia or parent/caregiver discomfort with outpatient management. As another balance measure, we used surveys to assess the percentage of patients/caregivers who felt fear, were not confident, or preferred inpatient management. We estimated the number of hospital days avoided due to outpatient management using 3.8 days as an estimated time for neutrophil recovery from the findings of our retrospective review.

### Analysis

**Fig. 3.**
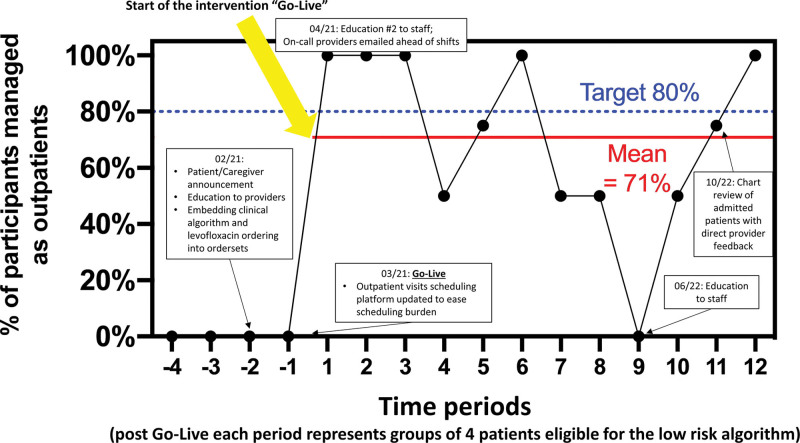
Run chart showing the change in participating patients with low-risk fever and neutropenia who received outpatient management for fever and neutropenia. Each data point represents four eligible patients.

We used a run chart to depict the change over time in the percentage of eligible patients managed initially as outpatients during the intervention. In the analysis, each data point represents four eligible patients (Fig. 3). We captured admission rates as a percentage during the implementation period. Descriptive statistics summarized survey data. This project was deemed QI and did not require a formal review by the Harvard Cancer Center institutional review board.

## RESULTS

From March 2021 to December 2022, our team managed 34 of 48 (71%) patients with solid tumors eligible for outpatient fever and neutropenia management according to the low-risk clinical algorithm (Fig. 3). There were 26 unique patients among these 34 patient encounters, as several patients had multiple encounters for fever and neutropenia. Sixty-four patients were ineligible according to the algorithm, with the most common reasons being signs or symptoms of septic shock (n = 21), high-risk neuroblastoma (n = 18), failed challenge of oral antibiotics (n = 8), or localized infection (n = 6).

For the balancing measures, nine of 34 patients (26.5%) initially managed outpatient were admitted before neutrophil count recovery. Reasons for admission included positive blood culture (n = 2), mucositis (n = 3), decreased oral intake, G-tube cellulitis, thrombocytopenia, and parent/caregiver discomfort with further outpatient management. The two patients admitted for bacteremia (*Streptococcus mitis* and *Lactobacillus rhamnosus*) presented to the clinic the next day after ER discharge. Notification occurred immediately upon blood culture results, and patients presented to the ER as hemodynamically stable. These patients did not require escalation of care beyond the oncology inpatient unit and remained without complications until discharge upon count recovery.

**Fig. 4.**
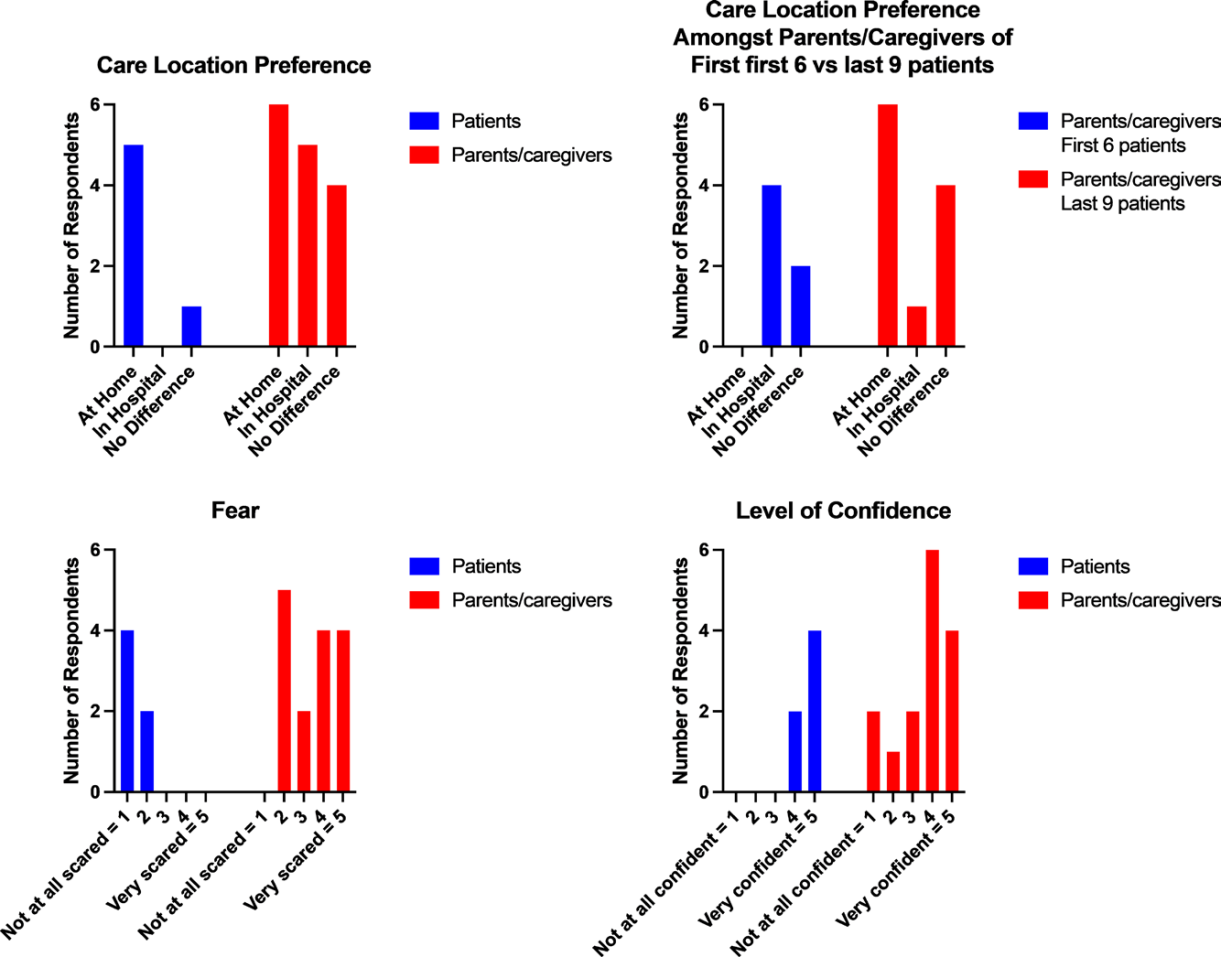
Results from surveys querying patients ≥12 years old and caregivers regarding preferences in care location and fear and confidence during their episodes of fever and neutropenia managed in the outpatient setting.

Regarding the patient and parents/caregiver preference survey, the response rate was 57.7% (15 of 26 unique patients) among parents/caregivers and 45.5% among patients (five of 11 patients aged 12 y or older) (Fig. 4). One patient filled out the survey after two separate episodes of fever and neutropenia and all other patients and parents/caregivers filled out the survey once. We counted both answers of the same patient in the survey results. Eighty-three percent (five of six patient responses) indicated a preference for the outpatient setting (home); one patient indicated no preference between settings. Among parents/caregivers, 40% (n = 6) preferred outpatient management, 33% (n = 5) preferred inpatient, and 27% (n = 4) had no preference. No patient expressed any of the three highest ratings on the Likert scale on the question exploring the fear of outpatient febrile neutropenia management versus 67% (n = 10) of parents/caregivers. For confidence level, 100% of patients chose the highest two confidence ratings with outpatient management versus 67% (n = 10) of parents/caregivers. Among parents/caregivers, of the first six respondents, four (67%) preferred hospital-based management and two (33%) expressed no preference. In contrast, among the subsequent nine caregiver respondents who received outpatient fever and neutropenia management after the algorithm was live for several months, only one (11%) preferred hospital-based management, two (22%) expressed no preference, and six (67%) preferred treatment at home.

Based on the time to neutrophil count recovery of 3.8 days and 25 patients receiving entirely outpatient management until neutrophil count recovery (nine of 36 were admitted), we avoided approximately 95 hospital days during the QI initiative.

## DISCUSSION

Outpatient management of febrile neutropenia for patients at low risk of bacteremia has yet to become the standard of care. Our work provides evidence that some patients with febrile neutropenia may avoid hospital days and receive home management safely. We managed 71% of low-risk outpatient patients. There are some potential contributing factors for not achieving the desired 80% aim. This project was a partnership with third-year fellows who ceased stewardship of the project upon graduation and had the least clinical responsibilities of the front-line providers.

Furthermore, there were a small number of patients eligible every month. Therefore, a considerable amount of time could elapse between a provider’s initial educational session and their first encounter with an eligible patient. Because there was no automatic trigger for the initiation of this algorithm, we relied on the education we provided and modifications in clinical decision support to ensure that providers would remember the algorithm. We did not explore an association between the parent’s/caregiver’s perception and the provider’s decision to pursue outpatient management. Still, it is possible that this also contributed to not meeting the desired goal. Although patients and parents/caregivers preferred outpatient management, it was more pronounced for patients than for parents/caregivers. We expected parents/caregivers to be hesitant about transitioning to outpatient management, which we interpreted from the survey questions about fear and confidence. Parents/caregivers expressed more fear and less confidence than patients. Notably, as the algorithm became more established, parents/caregivers expressed greater comfort with being managed at home, which was also expected given this significant practice change. In addition, we expected families to travel back within 24–48 hours for in-person follow-up until the count recovers. Given the long distances some patients travel, this expectation may have affected the caregiver/patient’s decision-making concerning outpatient versus inpatient management.

We anticipated the admission rate (26.7% of patients) after initial outpatient management. On review, the initial outpatient management for these patients did not affect their clinical course negatively. Compared with other published literature, the admission rate of 26.7% in this study was higher (Mullen et al,^8^ 14%; Paolino et al,^9^ 7%). These are relatively small sample sizes with insufficient statistical power to draw distinctions between admission rates. However, Dana-Farber/Boston Children’s Hospital Cancer and Blood Disorders Center is a tertiary care facility with many patient referrals from large distances, which may have contributed to the problem. Although the rate of bacteremia in both the retrospective review before Go-Live and during the QI initiative was around 10%, this only represents two to three patients in both cohorts, representing a potential sampling bias.

Although this project was successful, there are several limitations. The small sample size and the focus on patients with solid tumors may limit the generalizability of the results. In addition, the adherence rate to the algorithm was variable during the QI period. We did not observe special cause variation that would suggest the sustainability of the change. We did not longitudinally track the effect of increased outpatient visits. These could have affected clinic flow, other patients, and patients and families. Our approach requires close outpatient follow-up in the clinic and with patients and families. Therefore, sufficient outpatient infrastructure is necessary to manage these patients at home.

Similarly, manual data collection, extracted from the medical record and about each patient’s clinical course, was resource and time-intensive. The algorithm could not account for all clinical scenarios as there is still a lack of literature on the right cohort to manage as outpatients. As such, there were eligible patients for outpatient management admitted for sound medical reasons (eg, concerns for sepsis). However, we anticipated this finding, and it was part of the rationale for not setting the initial goal of achieving 100% management as outpatients. We did not consider the effect of antibiotic therapy in outpatient therapy, including *Clostridioides difficile* and resistance to levofloxacin. Although we solicited and incorporated parent/caregiver feedback, provided patient and parent/caregiver education, and distributed surveys after episodes of fever and neutropenia, we did not use a systematic, validated approach to fully assess the impact of our intervention on patients and parents/caregivers due to the nature of QI work. Therefore, we may not have captured all the unintended consequences of this practice change on patients and caregivers.

## CONCLUDING SUMMARY

Outpatient management of low-risk patients with febrile neutropenia is possible. Incorporating the patient’s and caregiver’s voices early on in QI interventions, especially those that entail a significant practice change that increases the caregiver’s responsibility in the home, is essential. The next steps for this initiative include addressing the program’s sustainability before expanding to other low-risk oncology patients who might benefit from fewer admissions to the hospital for fever and neutropenia.

## ACKNOWLEDGMENTS

The authors thank patients and their families for their partnership in this initiative. The authors appreciate all the time and sustained effort from the Jimmy Fund Clinic, including oncology nurse navigators, schedulers, and providers.

## Supplementary Material


