# Supplementary material for: Outpatient Management of Fever and Neutropenia in Low-risk Children with Solid Tumors: A Quality Improvement Initiative
Source: Pediatr Qual Saf. 2024 Sep 25;9(5):e771. doi: 10.1097/pq9.0000000000000771 (PMC11424129; doi:10.1097/pq9.0000000000000771)

Supplemental Figure 1

Primary ICD 10 code	Encounters
D70.8 - Other neutropenia	3
R50.9 – Fever unspecified	1
**Secondary Diagnosis**	**Encounters**
D70.8 – Other neutropenia	4
R50.81 – Fever presenting with conditions classified elsewhere	72
R50.9 – Fever unspecified	32

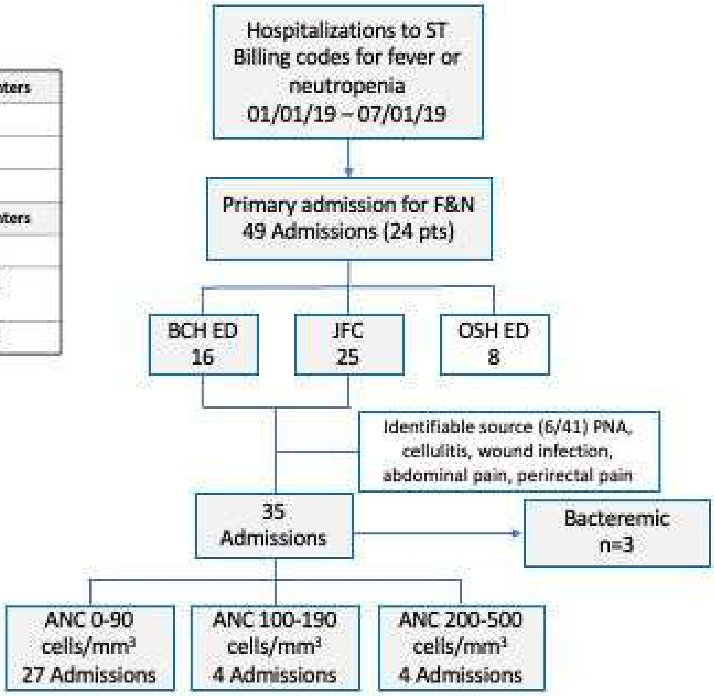

Supplement: Supplementary file 1 [file pqs-9-e771-s001.pdf]
