# Supplementary material for: Outpatient Management of Fever and Neutropenia in Low-risk Children with Solid Tumors: A Quality Improvement Initiative
Source: Pediatr Qual Saf. 2024 Sep 25;9(5):e771. doi: 10.1097/pq9.0000000000000771 (PMC11424129; doi:10.1097/pq9.0000000000000771)
Supplement: Supplementary file 2 [file pqs-9-e771-s002.pdf]

Outpatient Management of Fever and Neutropenia:
Low Risk Solid Tumor Oncology Patients Algorithm

Dana-Farber Cancer Institute | Boston Children's
Dana-Farber/Boston Children's Cancer and Blood Disorders Center

Definitions:
- Fever
  - ≥38.5C (preferably axillary) within last 24h
  - History of ≥38C x2 within last 24h
- Neutropenic: absolute neutrophil count (ANC) <500

Low Risk Patient Criteria:
1. Solid Tumor Malignancy, excluding: high-risk neuroblastoma, solid organ transplant, and neuro-oncology patients
2. Age >/= 12 months
3. Central access with a Port-a-Cath
4. Does not meet any other exclusion criteria
5. Patient/Family able to: follow-up in clinic within 24-48 hours, tolerate enteral Levofloxacin, and comply with discharge instructions

Exclusion Criteria:

Active Clinical Issue:
- Exhibiting signs or symptoms of septic shock (hypotension, hypothermia, tachypnea, hypoxia, altered mental status)
-High clinical or radiographic suspicion for neutropenic enterocolitis (typhlitis)
- >2 fluid boluses (40cc/kg) during initial evaluation
- Severe mucositis
- Inability to take or absorb enteral antibiotics (aversion, vomiting, severe diarrhea, etc)
-Evidence of significant localized infection (CVL, peri-rectal abscess, pneumonia, or cellulitis, etc)

Clinical Risk Factor:
- Hematological Malignancy, Central Nervous System Tumor, or post-Stem Cell Transplant
- High-risk Neuroblastoma
-Trisomy 21 Down syndrome patients
- Solid organ transplant patient
- Age < 12 months
- Receiving parenteral nutrition
- History of bacteremia in last 6 months
- Allergy or contraindication to Levofloxacin
- History of surgery in last 2 weeks
- External central access (PICC, Broviac) or implantable device (VP shunt, Ommaya, pain catheter, etc.) other than Port-a-cath. Patients with metal hardware (bone replacement) ***are eligible*** for outpatient management

Social Risk Factor:
- Unreliable social situation
- Speak language other than English, Mandarin, Arabic or Spanish
- First cycle of chemotherapy
- Patient unable to be evaluated at a BCH/South Shore ED/JFC

ED/JFC Discharge Criteria:
- Tolerated first dose of enteral Levofloxacin
- Received appropriate IV antibiotic after blood culture obtained
- Prescription for oral levofloxacin sent to a local pharmacy
- Oncology notified and in agreement

Fever in an Oncology Patient

→
- Follow existing fever and neutropenia guidelines and order-sets to administer appropriate initial IV antibiotics
- Notify Oncology after assessment and results

→ **Meets all LOW RISK criteria?**
- No → Admit patient to Oncology
- Yes → Give first dose of enteral Levofloxacin

→ **Patient tolerated Levofloxacin?**
- No → Admit patient to Oncology
- Yes →
  - Prescribe enteral Levofloxacin
  - Provide and review Patient/Family Education Sheet LINK

→ Yes → **Patient meets discharge criteria?**
- No → Admit patient to Oncology
- Yes →
  - Discharge patient home with levofloxacin in hand (if possible)
  - If in ED: Notify Oncology of Disposition

Oral/Enteral Levofloxacin Dosing
- <5 years: 10 mg/kg/dose twice daily for 7 days
- ≥5 years: 10 mg/kg/dose once daily for 7 days (maximum dose 750 mg/day)

Oncology arranges follow-up (see How-To-Guide) and notifies ST attending via page (if in ED)

→ **Patient's initial ED/JFC fever evaluation occurred Sun-Thur, or Friday by 3pm?**
- No →
  - Notify weekend on-call provider to ensure weekend check-in
  - Arrange for Monday JFC follow-up
- Yes → Arrange follow-up within 24-48 hours with labs/exam JFC visit

→
- Send QV notification to primary Oncology team, ST attending, and ST ONN using F&N template (weekend on-call team, if necessary)
- ST ONN arranges for daily call check-in and outpatient labs

→ **At first outpatient JFC visit: ANC still <500**
- No →
  - Discontinue antibiotics/daily labs/check-in
  - Notify primary Oncology Team of count recovery
- Yes → **Any NEW admission criteria*?**
  - Yes → Admit patient to Oncology
  - No →
    - ST ONN continues daily call check-in/outpatient labs
      AND
    - Arranges virtual follow-up

→ **ANC recovery to ≥500?**
- Yes →
  - Discontinue antibiotics/daily labs/check-in
  - Notify primary Oncology Team of count recovery
- No → **Any NEW admission criteria?***
  - Yes → Admit patient to Oncology
  - No →
    - Continue antibiotics
    - ST ONN continues daily check-in/outpatient labs

    → **> 7 days Neutropenic?**
    - No → (back to Continue antibiotics)
    - Yes → Notify primary Oncology Team to determine next steps

***New Admission Criteria:**
- Positive blood culture
- Inability to tolerate oral antibiotic
- Fever > 5 days
- Any signs of severe illness/concern for infection

**A patient receiving low-risk outpatient enteral antibiotic management who develops a new fever after 24 hours of being afebrile, will need to return to JFC/ED for re-evaluation
