# Supplementary material for: Outpatient Management of Fever and Neutropenia in Low-risk Children with Solid Tumors: A Quality Improvement Initiative
Source: Pediatr Qual Saf. 2024 Sep 25;9(5):e771. doi: 10.1097/pq9.0000000000000771 (PMC11424129; doi:10.1097/pq9.0000000000000771)
Supplement: Supplementary file 3 [file pqs-9-e771-s003.pdf]

Supplementary Table 1. Risk of bacteremia categorization for pediatric oncology patients to determine fever and neutropenia management

<table>
<tr><th>High Risk</th><th>Standard Risk</th><th>Low Risk</th></tr>
<tr><td>Exhibiting signs or symptoms of septic shock</td><td rowspan="7">Non-high risk patients that do not meet all low risk criteria</td><td>Solid Tumor Malignancy, excluding: high-risk neuroblastoma, solid organ transplant, and neuro-oncology patients</td></tr>
<tr><td>High clinical or radiographic suspicion for neutropenic enterocolitis (typhlitis)</td><td>Age >/= 12 months</td></tr>
<tr><td>Acute lymphoblastic leukemia (ALL) patients in all phases of therapy EXCEPT continuation (maintenance)</td><td>Central access with a Port-a-Cath</td></tr>
<tr><td>Relapsed ALL and relapsed acute myeloid leukemia (AML)</td><td>Patient/Family able to: follow-up in clinic within 24-48 hours, tolerate enteral Levofloxacin, and comply with discharge instructions</td></tr>
<tr><td>AML except APL post-induction</td><td rowspan="3">Does not meet any other exclusion criteria (Supplemental Figure 2)</td></tr>
<tr><td>Down syndrome patients with ANY oncology diagnosis in ANY phase of therapy</td></tr>
<tr><td>Advanced stage non-Hodgkin’s lymphoma (NHL), recurrent NHL, or recurrent Hodgkin’s disease treated with intensively myelosuppressive chemotherapy</td></tr>
</table>
